# Impact of mutations in the *mtrR*, *rpdlVD* and *rrl* genes on azithromycin resistance in *Neisseria gonorrhoeae*

**DOI:** 10.1371/journal.pone.0306695

**Published:** 2024-07-16

**Authors:** Florian Mauffrey, Fabrice Poncet, Damien Jacot, Gilbert Greub, Patrice Nordmann, Dominique S. Blanc

**Affiliations:** 1 Infection Prevention and Control Unit, Infectious Diseases Service, Lausanne University Hospital and University of Lausanne, Lausanne, Switzerland; 2 Swiss National Reference Center for Emerging Antibiotic Resistance, Fribourg, Switzerland; 3 Institute for Microbiology, Lausanne University Hospital and University of Lausanne, Lausanne, Switzerland; University of Tripoli, LIBYA

## Abstract

**Introduction:**

Bacterial sexually transmitted infections (STIs) pose a major public health problem. The emergence of antibiotic-resistant strains of *Neisseria gonorrhoeae* represents a serious threat to successful treatment and epidemiological control. The first extensively drug-resistant (XDR) strains (ceftriaxone-resistant and high-level azithromycin-resistant [HLR AZY]) have been reported.

**Aims:**

To identify molecular mechanisms implicated in azithromycin resistance in strains isolated from patients over a three-year period in a university hospital in Switzerland.

**Material and methods:**

From January 2020 to December 2022, 34 isolates (one per patient) were recovered from samples analyzed at the University Hospital of Lausanne. Eight genes involved in azithromycin resistance were sequenced: *mtrR* repressor (mtrCDE operon repressor) and his promotor *mtrR-pr*, *rplD* gene (L4 ribosomal protein), *rplV* gene (L22 ribosomal protein) and the four alleles of the *rrl* gene (23S rRNA).

**Results:**

With a cutoff value of 1 mg/L, 15 isolates were considered as being resistant to azithromycin, whereas the remaining 19 were susceptible. The C2597T mutation in 3 or 4 of the *rrl* allele confer a medium-level resistance to azithromycin (MIC = 16 mg/L, N = 2). The following mutations were significantly associated with MIC values ≥1 mg/L: the three mutations V125A, A147G, R157Q in the *rplD* gene (N = 10) and a substitution A->C in the *mtrR* promotor (N = 9). Specific mutations in the *mtrR* repressor and its promotor were observed in both susceptible and resistant isolates.

**Conclusions:**

Resistance to azithromycin was explained by the presence of mutations in many different copies of 23S RNA ribosomal genes and their regulatory genes. Other mutations, previously reported to be associated with azithromycin resistance, were documented in both susceptible and resistant isolates, suggesting they play little role, if any, in azithromycin resistance.

## Introduction

Sexually transmitted infections (STIs) pose a public health problem due to their frequency, risks of complications such as upper genital tract infections or congenital syphilis, long-term consequences like infertility, and the potential for HIV transmission. The emergence of multi-drug-resistant strains of *Neisseria gonorrhoeae* poses a serious threat to the treatment and control of the infection [1, 2]. The reduced susceptibility of these strains to third generation cephalosporins (C3G), which are the recommended first-line treatment, is particularly concerning. Worldwide, the emergence of multidrug-resistant strains has led to a therapeutic dead end [3].

Azithromycin is an antibiotic commonly used for the treatment of various bacterial infections, including those caused by *N*. *gonorrhoeae*. However, over time, *N*. *gonorrhoeae* has developed several mechanisms of resistance to azithromycin [4]. There are two main mechanisms through which *N*. *gonorrhoeae* can acquire resistance to azithromycin. The first one is linked to modifications of the antibiotic target site. Azithromycin binds to the 50S ribosomal subunit in bacteria, thus inhibiting protein synthesis. Some strains of *N*. *gonorrhoeae* can acquire mutations or modifications in the target site, specifically the 23S rRNA region of the 50S subunit, which reduces the binding affinity of azithromycin. Such mutations have been found to occur in the *rrl* genes, coding for the domain V of 23S rRNA [5, 6] and in ribosomal proteins L4 and L22 encoded by the *rplD* and *rplV* genes, respectively. The second mechanism concerns active efflux pumps. Bacterial cells often possess efflux pumps that actively pump out drugs, including antibiotics. Certain strains of *N*. *gonorrhoeae* can overexpress or upregulate efflux pumps, such as the MtrCDE pump, which can efficiently remove azithromycin from the bacterial cell [7–9]. This efflux mechanism effectively reduces the concentration of the drug within the cell, lowering its antibacterial activity and leading to resistance. Mutation in the *mtrR* promoter of the *mtr* gene and in the MtrR repressor gene have been reported to confer resistance to azithromycin. The presence of mosaic sequences in the *mtrR* genes, in the overlapping promoter regions for *mtrR* and *mtrCDE* and in the *mtrD* genes was also highlighted as a factor reducing azithromycin susceptibility in *N*. *gonorrhoeae* [10–12]. It is important to note that these mechanisms of resistance are not mutually exclusive, and *N*. *gonorrhoeae* strains can develop multiple mechanisms simultaneously, further reducing the effectiveness of azithromycin. The emergence of resistant strains highlights the need for ongoing surveillance, development of new treatment options, and implementation of effective strategies to combat the spread of drug-resistant *N*. *gonorrhoeae* infections. The objective of this study was to identify mutations implicated in azithromycin resistance in consecutive strains isolated from patients over a three-year period in our hospital. To do so, we analyzed the sequences of genes where mutations were reported to be responsible for the resistance to azithromycin.

## Material and methods

From January 1, 2020, to December 31, 2022, 34 *N*. *gonorrhoeae* isolates were recovered in our hospital (S1 Table in S1 File). Minimum inhibitory concentration (MIC) gradient strip tests were done for ceftriaxone, cefixime, azithromycin and ciprofloxacin according to manufacturer’s instructions. Breakpoints from the European Committee on Antimicrobial Susceptibility Testing (EUCAST) were applied. In addition, in agreement with previous publications [13–15], we considered five MIC categories: susceptible (≤0.25 mg/L), intermediate (>0.25–0.5), low-level resistance (1–2), medium-level resistance (4–32) and high-level resistance (≥64).

Following an overnight growth on chocolate plates in 5% CO_2_ atmosphere, DNA was extracted with the InstaGene Matrix kit according to the manufacturer’s instructions (Biorad, Hercules, California, USA). A literature review showed that 8 genes are frequently involved in the mechanism of resistance to azithromycin in *N*. *gonorrhoeae* (ref 1–9). These genes were amplified using primers and conditions already described (Table 1). Amplicons were sent to Microsynth (Balgach, Switzerland) for Sanger sequencing. Sequences were analyzed with BioNumerics^TM^ (version 8.1, available at http://www.applied-maths.com).

**Table 1:** Genes involved in azithromycin resistance mechanisms in *Neisseria gonorrhoeae* targeted in this study.

Gene	Function	Primers	Fragment length (bp)	References
*mtrR*-pr	mtrR promoter	MtrA-F:GCCAATCAACAGGCATTCTTA	401	[16]
		MtrA-R:GTTGGAACAACGCGTCAAAC		
*mtrR*	MtrR repressor	mtr2-F: GACCTGCTTCATAAGTGGA	1200	[13]
		mtr2-R:CAAGGCTTGATTATTTCCGG		
*rrl*	23S rDNA	NG23S1905-F:ACGGTCCTAAGGTAGCGA	863	[17]
		NG23S2769-R:TCTCATCTTCAGGCGAGTT		
*rrl1*	23S rDNA copy n°1	NG23S1F:GGCTATGAAGGCGGCGA	2400	[17]
		NG23SR:GAAGATGTGCAAGCATCGGA		
*rrl2*	23S rDNA copy n°2	NG23S2F:TTCAGATGAGTAATGTACACC	2400	[17]
		NG23SR:GAAGATGTGCAAGCATCGGA		
*rrl3*	23S rDNA copy n°3	NG23S3F: CAATCCGCAAGTCTGCCGA	2400	[17]
		NG23SR:GAAGATGTGCAAGCATCGGA		
*rrl4*	23S rDNA copy n°4	NG23S4F:CTCTCCGATCCCGAACTCG	2400	[17]
		NG23SR:GAAGATGTGCAAGCATCGGA		
*rplD*	Ribosomal protein L4	NGL4-F:CAGCGATGTTGTAGTTCGT	396	[17]
		NGL4-R2:TCAGAAACGACAGGCGCC		
*rplV*	Ribosomal protein L22	NGL22-F:TCAGCGACAATATGGTTGGT	621	[17]
		NGL22-R:AGCCCAGTCTTTAGTTACC		

The presence/absence of each of these mutations was reported in S2 Table in S1 File. Before conducting the analyses, this dataset was filtered. The *rplV* mutation was removed since no mutated allele was detected. Nara3854 and Nara3863 isolates appeared as outliers with a high MIC induced by *rrl1* mutations and were removed to ensure that any potential effects of other mutations would not be masked. Finally, *rrl1*-C2597T, *rrl2*-C2597T and *rrl4*-C2597T were also removed since no mutated alleles were present following Nara3854 and Nara3863 isolates removal.

Statistical analyses were performed using R version 4.3.1 and the MIC variable underwent a logarithmic transformation with a base of 2 prior to analyses. The correlation between presence of each mutation and the MIC value was tested using a binomial regression analysis. Moreover, for each mutation, the difference in MIC values between isolates harboring the mutation and those which did not was tested using a Wilcoxon test. This dataset was also used for a multivariate analysis. A Random Forest analysis was conducted (randomForest package v4.7–1.1) in order to reveal the most important predictors. Each isolate was categorized according to the MIC categories described above and this data was used as response variable in a classification model. Finally, a linear regression analysis was also conducted in order to test the correlation between the cumulated number of mutations for each isolate and the MIC value.

Data were obtained during a quality enhancement project at our institution. According to national law, the performance and publishing of the results of such a project can be done without asking the permission of the competent research ethics committee.

## Results

Phenotypic AMR results showed that 15 of the isolates (44%) were resistant to azithromycin, using the previous EUCAST clinical resistance breakpoint of MIC >0.5 mg/L. The most common resistance pattern was observed for ciprofloxacin (23/34, 68%). On the opposite, cefixime resistance was detected in only one (2.9%) of 34 isolates, and no resistance to ceftriaxone was observed. These data are comparable to the large EU and EEA study performed on 2018 isolates [15].

**Fig 1:**
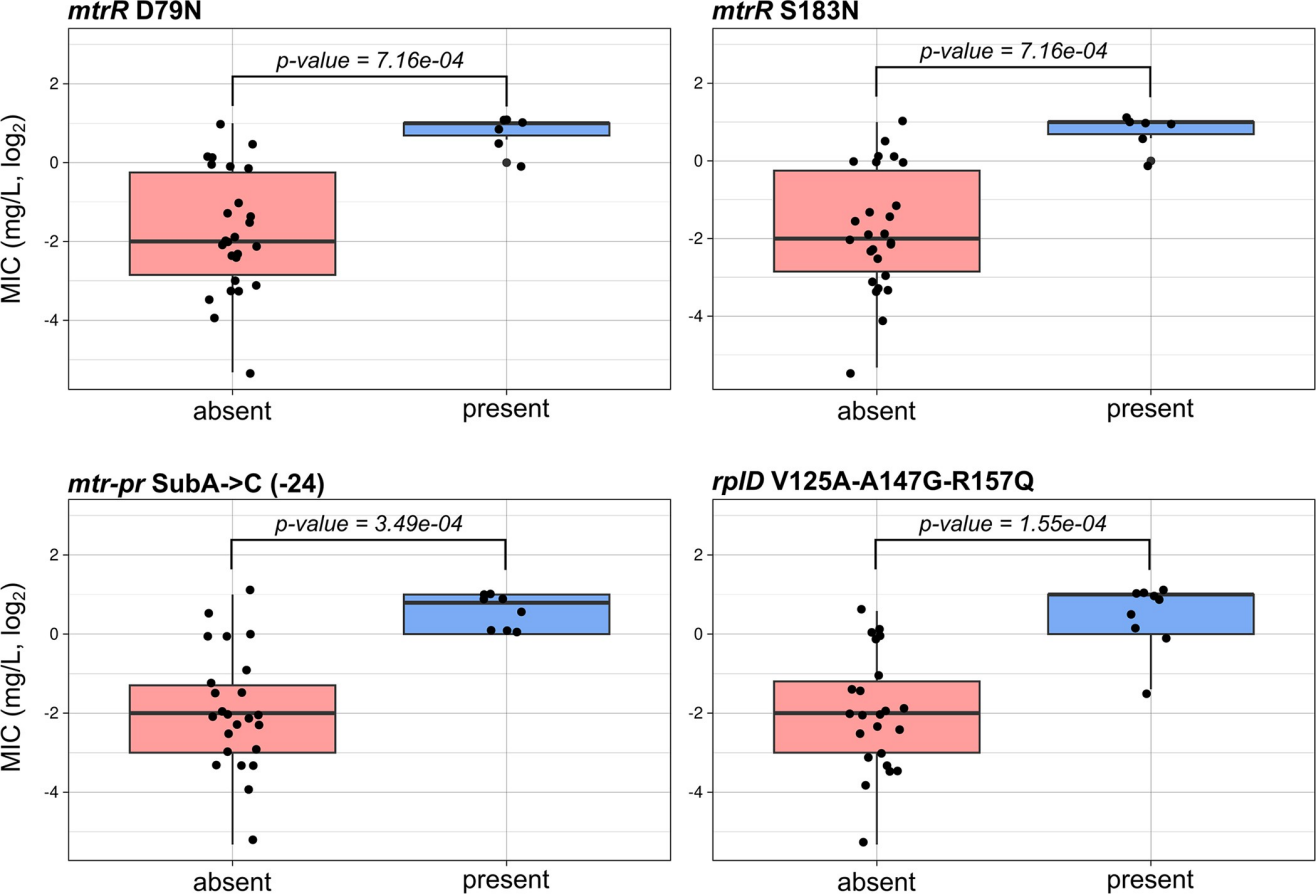
Boxplots illustrating MIC values for isolates with and without specific mutations. Only significant results are presented (Wilcoxon test p-value < 0.05).

**Fig 2:**
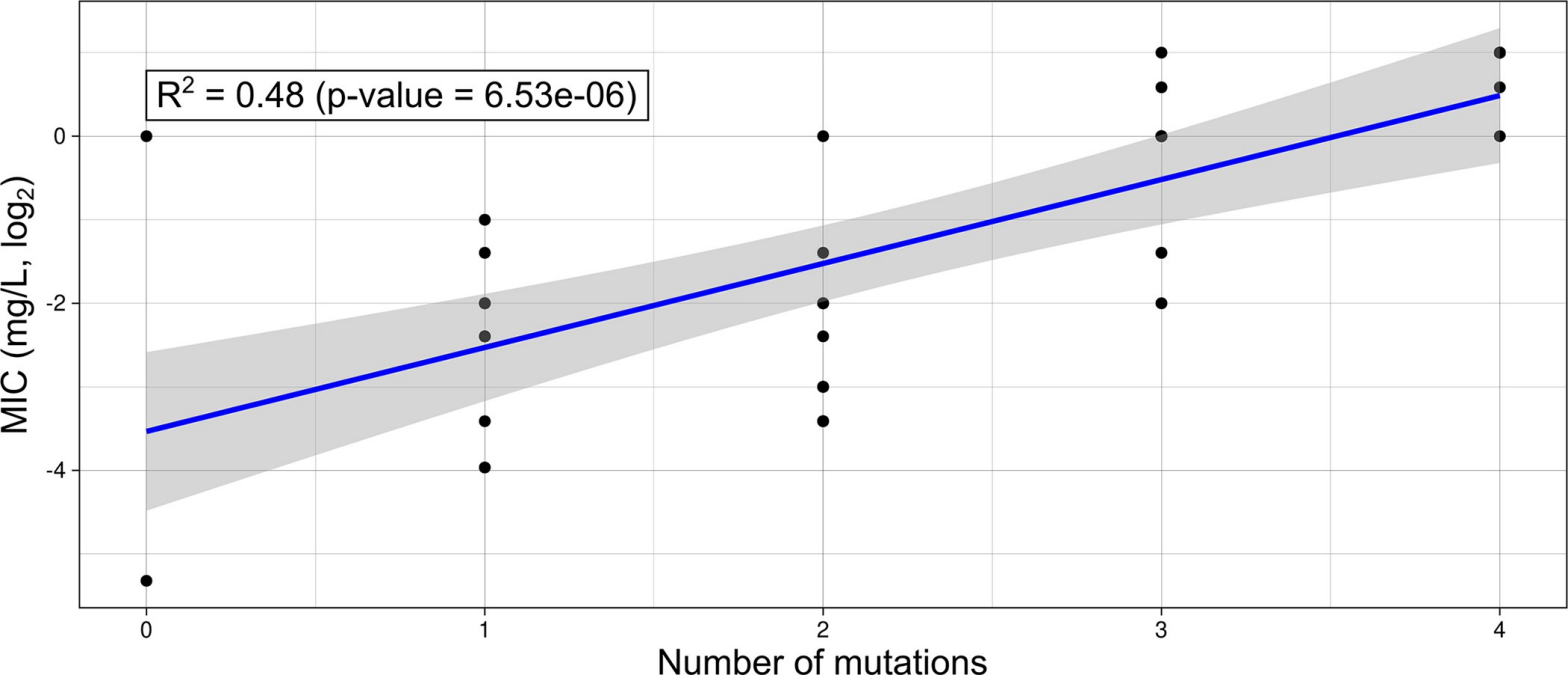
MIC value of each isolate depending on the cumulative number of mutations found among the studied genes. A linear regression analysis was conducted, and the regression curve is represented in blue. The correlation factor R^2^ and the p-value of the test are displayed.

Sequences of the 8 specific genes (Table 1) were obtained for the 34 *N*. *gonorrhoeae* isolates. The C2597T mutation in 3 or 4 of the *rrl* allele confer a medium-level resistance to azithromycin (MIC = 16 mg/L, N = 2). Univariate analyses showed that 4 mutations significantly affected the MIC values of the isolates: the three mutations V125A, A147G, R157Q in the *rplD* gene (N = 10), a substitution A->C in the *mtrR* promotor at position -24 (N = 9) and the 2 mutations D79N and S183N in the *mtrR* gene (N = 6, Fig 1). These mutations were significantly associated with MIC values ≥1 mg/L. These results were confirmed by the binomial regression analysis (S3 Table in S1 File). The random forest model had a good prediction power (Out-Of-Bag error = 18.25%) showing that MIC levels were well predicted by the mutations in these genes. The four previously cited mutations ranked as the four best predictors of the model (S4 Table in S1 File), with *rplD* V125A A147G R157Q and *mtr-pr* SubA->C (-24) outperforming the two *mtrR* mutations. The confusion matrix and model metrics showed that susceptible and low-level resistance categories were very well predicted while intermediate category had a F1 score of 0. This might be due either to the low number of isolates for this category or to a bad definition of the intermediate category. Indeed, most of these isolates were classified as susceptible. Finally, the number of mutations appeared to be significantly correlated with the MIC value with a correlation p-value < 0.001, meaning that mutations have a cumulative effect on azithromycin resistance and are complementary (Fig 2).

## Discussion

The *rrl* gene encodes the peptidyltransferase domain of the 23S rRNA, which is a component of the 50S ribosomal subunit. Azithromycin exerts its antibacterial effect by binding to the 50S ribosomal subunit, disrupting protein synthesis, and inhibiting bacterial growth. Specific mutations in the *rrl* gene can hinder the binding of azithromycin to the ribosome, reducing the drug’s efficacy and promoting resistance. This alteration disrupts the binding site of azithromycin to the 23S rRNA, preventing the drug from effectively inhibiting protein synthesis. As a result, the bacteria can continue to grow and multiply even in the presence of azithromycin, leading to resistance. The most common mutation associated with medium-level azithromycin resistance (4–32 mg/L) in *N*. *gonorrhoeae* is the C2597T mutation in the *rrl* gene [5, 13, 14, 18]. In our azithromycin-resistant *N*. *gonorrhoeae* isolates, the primary identified reason for a medium-level resistance is the presence of a single point mutation C2597T in three to four alleles of the *rrl* gene and is probably responsible for a low-level resistance (1–2 mg/L) in one isolate. However, a recent study showed that this mutation in only one *rrl* copy also found in susceptible isolates [15]. The A2045G mutations in the *rrl* genes can also result in high-level resistance to azithromycin [14]; this mutation was not observed in our isolates.

The second reason for resistance to azithromycin are mutations in the MtrCDE operon. This operon is a multidrug efflux pump system responsible for actively pumping out various antibiotics and toxic compounds from the bacterial cell, reducing their intracellular concentration and limiting their antibacterial effect. The MtrCDE operon consists of three genes: *mtrC*, *mtrD*, and *mtrE*, which encode proteins involved in the efflux pump’s function. The *mtrR* gene encodes the MtrR protein acting as a repressor on the expression of the mtrCDE operon. This repressor is positively regulated by the *mtrR* promotor. Azithromycin resistance is driven by the overexpression of the efflux pump MtrCDE. The primary mutation in the MtrCDE operon conferring resistance to azithromycin is a substitution A->C in the *mtrR* promoter at position -24. This mutation results in an increased MtrCDE efflux of substrate antimicrobials, e.g. macrolides and b-lactam antimicrobials [18]. In our isolates, this substitution was only found in resistant isolates, confirming the role of this mutation in azithromycin resistance. However, the *delA* mutation in the 13bp inverted repeat, which was also described as involved the resistance [14, 18, 19], was found both in susceptible and resistant isolates. Mutations in the MtrR protein were also described to impact its regulatory function and contribute to the increased activity of the MtrCDE efflux pump. In our isolates, the D79N and S183N substitutions were found only in resistant isolates, suggesting their implication. However, other mutations reported as possibly involved in resistance (A39T, A46T, GD45D, H105Y, R44H, T86A, Y48D) [14, 18, 19] were not found to be statistically associated with resistance in our isolates. The G45D mutation in the *mtrR* gene has been reported to confer intermediate resistance (MIC >0.25 - <0.5) to azithromycin [14, 18]. Interestingly, all our isolates with this mutation showed an MIC between 0.19 and 0.5 mg/L, suggesting the role of this mutation in a decrease susceptibility to azithromycin.

The third mechanism of resistance to azithromycin is mutation in the *rplD* gene. The *rplD* gene encodes for the ribosomal protein L4, which is a part of the 50S subunit of the bacterial ribosome. Azithromycin resistance resulting from mutations in the *rplD* gene is primarily associated with structural modifications in the ribosomal protein L4. The G70D mutation in the *rplD* gene has been the most frequently reported in existing literature. Nevertheless, recent studies [8, 15, 20] have refuted its involvement in azithromycin resistance. This mutation was not identified in any of our isolates.

The mutations V125A, A147G, and R157Q in the *rplD* gene have been documented in only three publications. In a French study, these mutations were detected in four azithromycin-intermediate isolate and one azithromycin-resistant isolate [13]. In a Canadian survey, 92.9% of isolates carried these mutations but the authors did not confirm any association with resistance [21]. In a European survey, these mutations were found in 26 isolates but were not linked to an increased MIC of azithromycin [22]. Interestingly, in our study, these mutations were among the strongest predictors in our random forest model, suggesting their potential role in azithromycin resistance. It remains unclear in studies investigating *rplD* mutations whether these mutations were not discovered or were not actively sought after. Further investigations are required to definitively determine their involvement in this resistance.

A limitation of our study lies in the absence of an analysis of the mosaic structure within the *mtr* operon. This is significant because mosaic multiple transferable resistance (*mtr*) efflux pump alleles, arising from horizontal gene transfer from various *Neisseria* species, have been associated with increased azithromycin resistance [12, 15]. This phenomenon, in conjunction with identified mutations or not, could be at the origin of decreased susceptibility to azithromycin. Further investigations would be necessary to disentangle the respective contributions of these mutations and *mtr* mosaic alleles to the observed MIC. The interplay of these resistance mechanisms can vary among different strains of *N*. *gonorrhoeae*, leading to different levels of resistance. Moreover, some strains may possess multiple mechanisms simultaneously, making them highly resistant to azithromycin and other antibiotics.

To address the challenge of azithromycin resistance in *N*. *gonorrhoeae*, ongoing surveillance of antibiotic resistance is crucial, as well as the development of new treatment strategies and public health measures to prevent the spread of resistant strains.

## Supporting information

S1 File Supplementary Tables S1 to S4. (XLSX)
